# Long-term effects of interference on short-term memory performance in the rat

**DOI:** 10.1371/journal.pone.0173834

**Published:** 2017-03-13

**Authors:** Mégane Missaire, Nicolas Fraize, Mickaël Antoine Joseph, Al Mahdy Hamieh, Régis Parmentier, Aline Marighetto, Paul Antoine Salin, Gaël Malleret

**Affiliations:** 1 Forgetting and Cortical Dynamics Team, Lyon Neuroscience Research Center (CRNL), University Lyon 1, Lyon, France; 2 Centre National de la Recherche Scientifique (CNRS), Lyon, France; 3 Institut National de la Santé et de la Recherche Médicale (INSERM), Lyon, France; 4 Neurocentre Magendie, INSERM U1215, Université de Bordeaux, Bordeaux, France; Tokai University, JAPAN

## Abstract

A distinction has always been made between long-term and short-term memory (also now called working memory, WM). The obvious difference between these two kinds of memory concerns the duration of information storage: information is supposedly transiently stored in WM while it is considered durably consolidated into long-term memory. It is well acknowledged that the content of WM is erased and reset after a short time, to prevent irrelevant information from proactively interfering with newly stored information. In the present study, we used typical WM radial maze tasks to question the brief lifespan of spatial WM content in rodents. Groups of rats were submitted to one of two different WM tasks in a radial maze: a WM task involving the repetitive presentation of a same pair of arms expected to induce a high level of proactive interference (PI) (HIWM task), or a task using a different pair in each trial expected to induce a low level of PI (LIWM task). Performance was effectively lower in the HIWM group than in LIWM in the final trial of each training session, indicative of a “within-session/short-term” PI effect. However, we also observed a different “between-session/long-term” PI effect between the two groups: while performance of LIWM trained rats remained stable over days, the performance of HIWM rats dropped after 10 days of training, and this impairment was visible from the very first trial of the day, hence not attributable to within-session PI. We also showed that a 24 hour-gap across training sessions known to allow consolidation processes to unfold, was a necessary and sufficient condition for the long-term PI effect to occur. These findings suggest that in the HIWM task, WM content was not entirely reset between training sessions and that, in specific conditions, WM content can outlast its purpose by being stored more permanently, generating a long-term deleterious effect of PI. The alternative explanation is that WM content could be transferred and stored more permanently in an intermediary form or memory between WM and long-term memory.

## Introduction

That memory is not a unitary process is now well accepted and several forms of memory have been described in the literature [1–3] and can be traced back to the antiquity [4]. In the late 19^th^ century, Ebbinghaus developed the first scientific approach to study memory. By using himself as a subject, Ebbinghaus carried out a series of experiments during which he memorized lists of three-letter nonsense syllables [5]. Ebbinghaus clearly observed that he initially forgot an important quantity of items rather rapidly, in the first few minutes after their initial learning, but that after one hour the remaining learned items could be steadily conserved for several weeks. Such work surely inspired James [6] that divided memory into primary and secondary memory—one fragile and the other more stable—triggering the distinction still used nowadays between short-term and long-term memory. In 1968, Atkinson and Shiffrin described short-term memory as a simple storage system through which information would transit before being eventually transferred into a more durable long-term memory [7]. However, this simple storage model did not explain how we can use and manipulate information stored in short-term memory to perform cognitive tasks such as mental calculation. It is to solve this issue that the term "working memory" was coined by Miller, Galenter and Pribram [8] based on the idea that the mind functions like a computer [4], a prevalent analogy in the 1960’s. These authors defined WM as a quick-access memory used to execute a certain plan of actions, guiding our behavior according to our general knowledge of the world. Nevertheless, WM and short-term memory have rarely been considered independent and the two terms have often been used synonymously [9] (however see [10]).

In human cognitive research, several models were designed to explain WM functioning, such as the multicomponent model of Baddeley [11] or Cowan's Embedded Process theory [9]. All these models see WM as having limited capacity in terms of items stored (the maximum is assumed to be 4 [11,12] or 7 [13]) and in terms of duration, with a retention interval being "*brief*" [14]. This time limit is not precise but is generally in the order of seconds. Cowan thus found that the memory for a tone collapsed after 5 to 10 seconds [15]. Despite this dogma, new models of WM have emerged these past few years, in particular to question its limited capacity regarding the number of items stored [16] but also concerning its duration [17–19]. However, the limited capacity of WM is certainly adaptive if we consider that information temporarily stored should be forgotten in order not to overload our brain with irrelevant information [20,21]. Adaptive forgetting is thus inherent to WM [22]. According to the core definition of WM, its content is only stored temporarily. If information in WM Is only stored temporarily, that means that such information should logically be no longer stored and therefore forgotten (unless of course this information is being transferred into long-term memory, but in that case we can no longer speak of WM). Previous work in human subjects have revealed that WM is particularly sensitive to the negative effect of proactive interference (PI) [23], which are the information previously stored that can interfere proactively with later WM performance [24,25].

In animal behavioral neuroscience, the term "working memory" has first been used by Olton [26] to characterize “*the ability of an animal to keep track of its location in space by remembering where it has been*”. Rats were thus trained in a radial maze to find food rewards at the end of the arms or alleys of the maze. In these conditions, the strategy used by the rat to optimize its search for food is to prevent its reentry into a previously visited arm. Such reentry causes a non-rewarded action as food reward has already been collected. This alternation or “non-match” strategy is at the base of numerous delayed-non-match-to-place (DNMTP) or delayed-non-match-to-sample tasks still used nowadays to assess WM in different species such as rodents, birds, or human and non-human primates [17,27–33]. In these tasks, the subject is required to temporarily store information concerning a previously presented place or item during a “sample phase” in order to choose, usually after a short delay, an alternate option during a subsequent “choice phase”. Olton (1978) suggested that memory for arms visited is held within a limited-capacity WM that may be reset at the end of each trial by deleting its content; this way, PI from a recently completed trial does not interfere with retention of events within a subsequent trial. However, when Roberts and Dale reinterpreted these data in 1981, these authors actually found a PI effect which was originally masked by the strategy adopted by the rats of circling the maze [34]. In their experiments, Roberts and Dale showed that PI—information related to past trials—impaired performances during the course of a single day of training (being composed of several trials) in the radial maze. In their experiments, the delay between trials was of 60 to 240 seconds during which information from a trial n was no longer forgotten, interfering with performance on a trial n+1. These durations exceed the classically assumed time limit in the order of seconds after which the content of WM is supposed to be forgotten. However, Roberts and Dale found that when rats were trained for several days with several trials per day, and even if the performances dropped across a single daily session of trials, rats’ performance globally improved over days. Therefore the content of WM would be forgotten during the 24h-delay between two days, suggesting that the duration limit of WM is inferior to 24h.

Here, we question the duration capacity of WM, and therefore the possibility to discriminate it from long-term memory, by showing that WM can persist in time, or alternatively that WM content can be transferred into an intermediary memory form between WM and long-term memory. We used two delayed-non-match-to-place WM radial maze tasks designed in our laboratory [28,35,36], and we showed that when the repetitiveness of the task is increased (compared to the task used by Roberts and Dale), the content of WM can be kept in memory for days (either in WM or another form of memory), impairing rats' performance after several days of training by generating long-term PI. In fact, we show that after several days of training, WM reset is no longer possible. Moreover, we dissected the PI effect by examining the effect of spaced versus massed training, but also by revealing the crucial role of spatial pattern separation in the building of PI.

## Materials and methods

### Experiment 1

#### Subjects

A total of 73 Dark Agouti rats aged of 10 weeks (200-250g) were purchased from Janvier, France. They were housed in individual cages with a 12h/12h (9am-9pm) light/dark cycle with *ad libitum* access to food and water. Rats were food deprived so that they were at 85% of their free-feeding weight during the whole behavioral procedures in the radial maze. This study was carried out in strict accordance with the recommendations of the Lyon1 University (CE2A-UCBL 55) and the European (2010/63/EU) ethical committees for the use of experimental animals. The protocol was approved by the Lyon1 University ethical committee for the use of experimental animals (Permit Number: DR2013-20). All efforts were made to minimize suffering.

#### Behavioral apparatus

**Fig 1:**
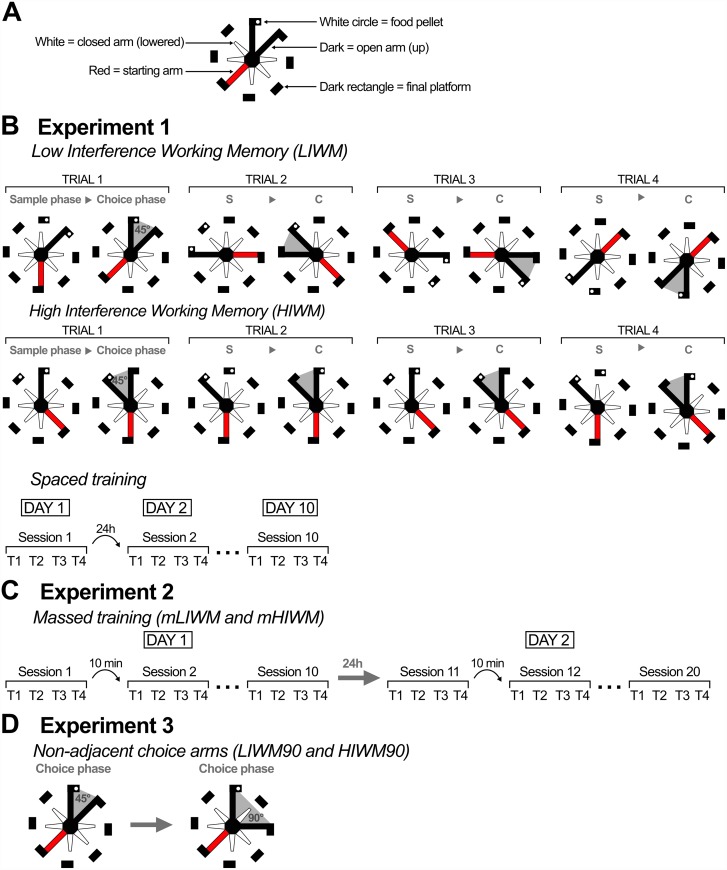
Behavioral protocols used in Experiments 1 to 3. (A) Drawing code used to represent the radial maze. (B) Classic LIWM and HIWM training (see also Materials and methods) performed in Experiment 1, illustrated with one session (four trials). The pair of arms used for each trial is different for the LIWM training while it is identical for HIWM training. Training was spaced, meaning that two sessions were separated by 24h with a total of 10 sessions. The sequence of arms presentation shown in (B) is only informative and does not represent the sequence used each day. This sequence is different every day and pseudo-randomly determined by the experimenter. (C) In Experiment 2, the two tasks used were the same as in A), except that training was massed (mLIWM and mHIWM): each session was separated by 10 min, with 10 sessions on day 1 and 10 other sessions on day 2. (D) In Experiment 3, we used a spaced training as in A), but the arms used during the choice phase were no longer adjacent but formed a 90° angle (LIWM90 and HIWM90), as illustrated in the example.

The behavioral apparatus used for behavioral tasks was an elevated eight-arm radial maze [35,36]. The eight arms (65 cm long x 12 cm wide) started from one octagonal central platform (33 cm diameter) and ended on eight rectangular platforms (17 cm x 25 cm). The experimenter could automatically move each arm either in an upward (open) or downward (closed) position, while monitoring rat movements from an adjacent room using a video camera above the maze (Fig 1A). The closed position of an arm prevented the rat from accessing the platform at its end. On each of these eight platforms, a square food well (2 x 2 cm and 0.5 cm deep) could be filled with odorless food rewards (Dustless Precision Pellets; Bioserve, Frenchtown, NJ) invisible for the rat from the central platform. The maze was located in a room with a number of distal visual cues (e.g., door, furniture…) allowing animals to use spatial (allocentric based [37–39] hippocampus-dependent [40–42]) memory to remember the position of arms already visited. All rats' movements in the maze were video-recorded for off-line examination.

#### Behavioral protocol

Food deprived rats underwent a 7-day habituation period during which they became accustomed to the radial maze environment and learned to collect food rewards in the platform wells. After this habituation period, rats were divided in two groups, trained in one of the two WM tasks described below (Fig 1B). These tasks are based on a delay non-match to place (DNMTP) paradigm commonly used to test WM in both primates and rodents. All tasks were run between 10 am and 1 pm during the light phase of the rats 24h-cycle.

- Low interference working memory (LIWM) task
  Rats were submitted to one session of four trials per day during 10 days, so that the between-session delay was 24h. One trial was composed of a sample and a choice phase. In the sample phase, the rat started from a pseudo-randomly chosen platform and was forced to enter one pseudo-randomly chosen baited arm/platform with all other arms being lowered (closed). Once the rat collected the reward, it was put back to a transfer cage adjacent to the maze for a short delay of 15 seconds (within-trial delay). During the subsequent choice phase, the rat had the choice to enter either the arm that had just been visited and empty of food, or an adjacent novel arm (right or left chosen pseudo-randomly) leading to a second food reward. A positive score was attributed to the rat in case of the choice of the novel arm, according to the DNMTP paradigm: the rat thus had to use its WM to remember the arm already visited during the sample phase to choose the other one during the choice phase. During the 15 second-delay between two trials (between-trial delay), the rat was placed in the same transfer cage as the one used during the within-trial delay. The particularity of the LIWM task is that different pairs of arms were used for each trial of a given session (4 trials given in 4 different pairs of arms = 8 arms of the maze).
- High interference working memory (HIWM) task
  The protocol of the HIWM task is exactly the same as in the LIWM task described above, except that the same pair of arms is used for each trial of the 10 sessions in total. In this very repetitive task, the level of proactive interference is very high between trials. We showed that this task relies on forgetting of past trials for the subject to succeed on an ongoing one [29].

### Experiment 2

#### Subjects and behavioral apparatus

Twenty eight new Dark Agouti rats were used in this experiment and were housed and handled like the ones from experiment 1.

#### Behavioral protocol

The protocol was similar to the one in experiment 1 with two groups of rats being trained in the LIWM or the HIWM task. However, spaced training used in experiment 1 was replaced by a massed training (mLIWM and mHIWM): rats were trained in 10 sessions per day during 2 days, with a between-session interval of 10 minutes and not 24h as in experiment 1 (Fig 1C). Therefore, these rats were trained for 20 sessions in total. However, the delay between session 10 of day 1 and session 1 of day 2 (session 11) was 24h.

### Experiment 3

#### Subjects and behavioral apparatus

Twenty six new Dark Agouti rats were used in this experiment and were housed and handled like the ones from experiment 1.

#### Behavioral protocol

The protocol used in experiment 3 was similar to the one used in experiment 1 (with spaced training). However, while in experiment 1 the sample arm and the choice arm for a given trial were adjacent (forming a 45° angle), in experiment 3 they were separated by another arm (each pair of arms forming a 90° angle: LIWM90 and HIWM90) (Fig 1D). In consequence, the spatial overlap between extra-maze visual cues used by the rats to discriminate the sample and the choice arm in these conditions was smaller, lowering the need for pattern separation in these tasks [43].

### Statistical analyses

Behavioral data (performance scores expressed as a percentage of correct choices) were analyzed using two-way ANOVAs (Analysis of Variance) for repeated measures with Block (2 sessions of training), Trial (1 to 4) and Group (LIWM versus HIWM trained rats) as main factors (PRISM GraphPad Software version 6). Further comparisons were performed by *post hoc* (Sidak's multiple comparison test and split-by Group one-way ANOVA) analyses for particular within-group comparisons. Data are expressed as means ± s.e.m.

## Results

### Experiment 1

**Fig 2:**
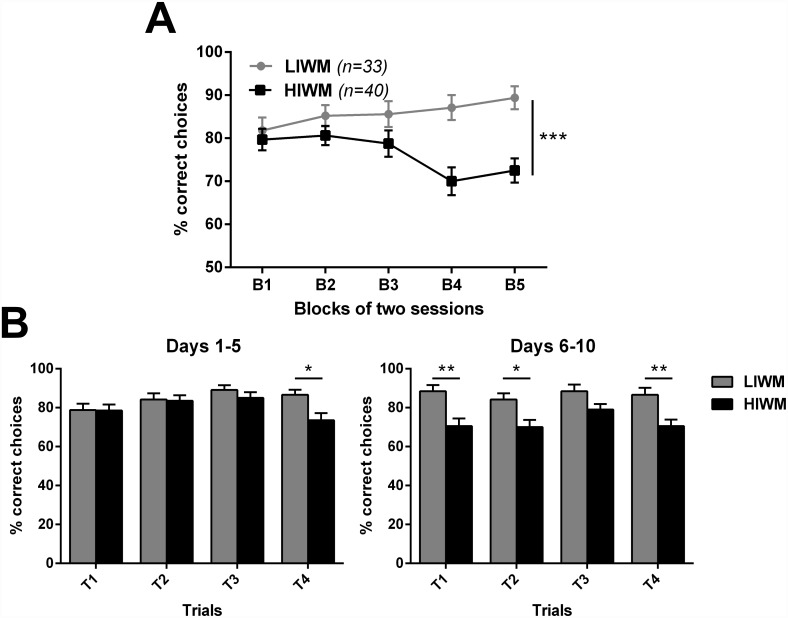
Performance drop in the final sessions of HIWM training reveals accumulation of PI across days, and hence long-lasting WM. (A) Evolution of the percentage of correct choices across blocks of two sessions, for rats trained in the LIWM or the HIWM task. ANOVA revealed a significant Group effect (*** p < 0.001), due to the drop of performance in the HIWM group across days revealed by the *split-by* Group analysis. (B) Percentage of correct choices made by trial for the first and the last 5 days of training, for the two groups. For Days 1 to 5 ANOVA revealed no Group effect but a significant Trial effect with a drop of performance on trial 4 for the HIWM group (Sidak's *post-hoc* test, * p < 0.05), indicative of short-term PI. On the contrary for days 6 to 10 there was a significant Group effect, due to lower performance in the HIWM group from the very first trial (Sidak's *post-hoc* test, ** p < 0.01). This reduction of performance in the HIWM task could not be attributable to short-term PI, but to accumulation of PI across days indicating that WM was not entirely reset at the end of each training and could partly last at least 24h.

Fig 2A shows the evolution of performance (expressed as the percentage of correct choices averaged by blocks of two sessions each) across the ten days of spaced training in the LIWM or the HIWM tasks. Performance of rats trained in the HIWM task was clearly inferior to the one of LIWM trained rats. ANOVAs revealed a significant Group effect [F (1, 71) = 12.10; p = 0.0009], a significant Group x Block interaction [F (4, 284) = 4.725; p = 0.0010], but no significant Block effect [F (4, 284) = 1.058; p = 0.3776]. *Post-hoc* split-by Group analyses also revealed that HIWM rats significantly decreased their performance over time [F (3.733, 145.6) = 4.260; p = 0.0034] likely due to the accumulation of PI after several days of training in this task. Performance of HIWM rats was thus significantly inferior to the one of LIWM rats in the final training blocks 4 and 5 (70 ± 3.221 vs 87.121 ± 2.903 on block 4 and 72.5 ± 2.802 vs 89.394 ± 2.672 on block 5, p = 0.0001 for both blocks).

Our next aim was to see whether rats’ performance in WM declines from trial 1 to trial 4 (“within-session/short-term” PI effect as classically described) but always returns to errorless performance on the first trial of the next day, a process known as resetting and defined as the capacity to erase or “reset” the contents of WM at the end of a given session [26,34]. We thus analyzed rats’ performance by trial rather than by day over the first (Day 1–5) and last 5 days (Day 6–10) of the experiment (Fig 2B). We summed the number of correct choices made for each one of the 4 trials during 1) the five first sessions (days) when the effect of PI (i.e. difference between the LIWM and HIWM training conditions) was not yet significant as shown in Fig 2A, and during 2) the five last sessions when the effect of PI was clear. At the beginning of training (days 1 to 5), no significant Group effect but a significant Trial effect [F (3, 213) = 4.049; p = 0.0079] was found. *Post hoc* analyses revealed that performance of rats trained in the HIWM task was lower on Trial 4 as compared to LIWM trained rats (p = 0.0109). This indicates the existence of a within-session PI effect, with PI being accumulated throughout a given session. However, resetting of WM was seemingly effective as performance of HIWM trained rats returned to the level of performance of LIWM trained rats on trial 1 of these first five days (p > 0.9999). No between-session effect of PI was thus observed during this stage of training. In contrast, when analyzing the performance by trials at the end of training (days 6 to 10), ANOVAs revealed a significant Group effect [F (1, 71) = 18.35; p < 0.0001], with performance of HIWM trained rats being lower than performance of LIWM trained rats from the very first trial (T1) of the day (p = 0.0011). This result indicates that, after a certain amount of training, resetting was not as effective for rats trained in the HIWM task, suggesting that information stored in WM (supposedly for 15 seconds) was not forgotten but instead was consolidated into a more durable form of memory and could therefore interfere with subsequent WM functioning in the following days of training in the same task.

### Experiment 2

**Fig 3:**
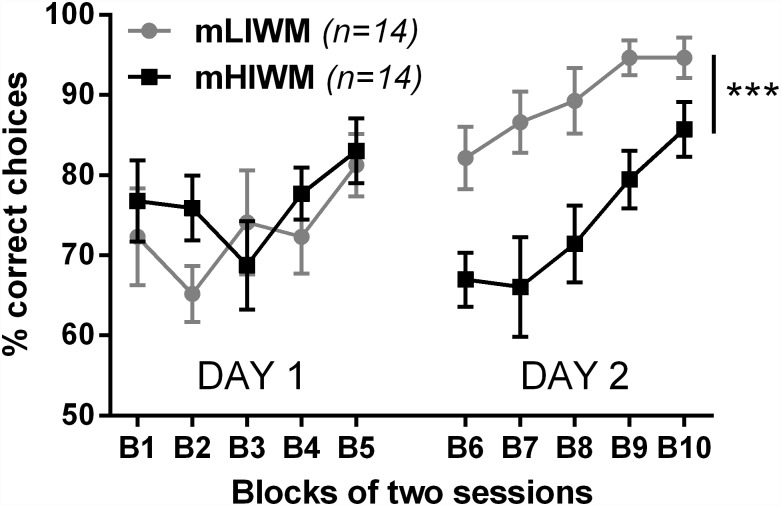
Massed training prevents performance drop in the HIWM group but long-term proactive interference appears after a 24 hour-interruption that allows consolidation of the content of WM into a more durable form of memory. Percentage of correct choices across blocks of two sessions with a massed training of 10 sessions by day during two days. ANOVA revealed a significant Group effect only on day 2 (*** p < 0.001) due to lower performance in the HIWM group. A *split-by* Group analysis of performance on day 2 showed a significant improvement in HIWM trained animals leading to similar level of performance between the two groups on final block 10.

To provide further support to the suggestion that WM can generate long-term PI effect by being consolidated into a more durable form of memory, we attempted to demonstrate that 24 hour-spaced WM training was necessary to observe “between-sessions” PI as found in experiment 1. Indeed, since the famous work of Ebbinghaus [5], spaced training has been known to be superior to massed training when it comes to consolidate information into long-term memory. This superiority has been confirmed since then in many fields of memory, from learning concepts to motor skills, and from invertebrates to humans (for review see [44]). Hence, if the building of PI from one day to another that we observed in the HIWM task in experiment 1 was due to consolidation of WM into a more durable form of memory during the 24h between-session interval, then the long-term PI effect should disappear if the between-session gap was diminished to an interval shorter than needed for consolidation. We thus trained a new group of rats with a massed training in the LIWM and HIWM tasks (here called mLIWM and mHIWM), during which the between-session interval was shortened from 24h to 10 minutes. We kept the same number of sessions as in the spaced training, meaning that rats underwent 10 sessions in one single day. Strikingly, massed training abolished the drop of performance previously observed after Block 3 (6 sessions) in the HIWM task (Fig 3 –Day 1): ANOVA revealed no significant Group effect over 5 blocks (10 sessions) of training massed in a single day [F (1, 26) = 0.7520; p = 0.3938]. Thus, spaced training is a necessary condition for PI to accumulate between training sessions and impair rats' performance in the HIWM task. This finding suggests that long-term PI observed in experiment 1 was due to some consolidation of the content of WM into a more durable form of memory during the between-day interval, as further confirmed by results obtained after a 24h interruption of massed training (i.e. in Day 2—see below).

Having found that spaced training was a necessary condition for the appearance of long-term PI, we then examined whether a 24h interruption of training, compatible with consolidation processes, would be a sufficient condition for the long-term PI effect to appear. In order to see if a PI effect would appear after a 24h interruption of training, we kept training the rats for a second day of 10 additional sessions (again with 10 minutes delay). Strikingly, PI appeared on this second day and induced a performance drop for rats trained in the mHIWM task compared to rats trained in the mLIWM task (Fig 3 –Day 2): ANOVAs revealed a significant Group effect [F (1, 26) = 20.81; p = 0.0001]. This drop was visible from the first block of the second day (p = 0.0361), showing that PI from memories relative to the first day of training (i.e. long-term PI) was now altering WM function. Thus, a 24h interruption of training, allowing memory consolidation, is a sufficient condition for the appearance of long-term PI effect after certain amount of training in the HIWM task. However, the reduction of performance of the HIWM group seen in the second day of massed training was transient. ANOVAs thus revealed a significant effect of Block in day 2 [F (4, 104) = 7.496; p < 0.0001], which was essentially due to a significant improvement of performance of the HIWM group, as revealed by a *split-by* Group one-way ANOVA [F (2.673, 34.75) = 4.931; p = 0.0075]. Sidak's multiple comparisons test indicated that performance on final Block 10 was not significantly different between LIWM and HIWM rats (p = 0.4462). Thus, during massed HIWM training, not only no deleterious effect of repeated testing within a day was observed, but performance levels even augmented upon repetitive training sessions in day 2, a result suggesting the recruitment of potential mechanisms counteracting PI during massed training. In summary, experiment 2 shows that spaced training is a necessary condition for the appearance of a long-term PI effect after a certain amount of training in the HIWM task, further supporting the idea that under certain circumstances, the content of WM can be consolidated into a more durable form of memory.

### Experiment 3

**Fig 4:**
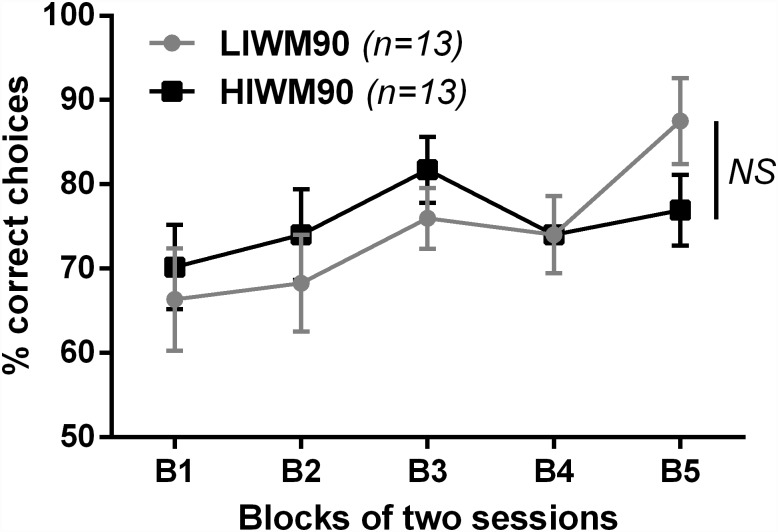
Reducing the need for pattern separation prevents performance drop in the HIWM group. Percentage of correct choices across blocks of two sessions with a spaced training in the two tasks during which the angle between two choice arms was 90° to reduce the need for pattern separation relative to the same tasks with 45° spaced-arms as used in experiment 1. ANOVA revealed no Group effect (NS: non-significant).

Finally, in experiment 3 our goal was to identify one potential cause of long-term PI building. Based on previous findings from our team showing a specific silencing of neuronal activity in the DG after HIWM training (identical to experiment 1) [28], we expected that engagement of the pattern separation function of the DG could be involved in the accumulation of PI that causes the performance drop seen in the HIWM task. Indeed, one of the essential functions of the DG is to perform pattern separation in order to differentiate similar memories by making their neuronal representation more distinct [45]. As we previously showed that the DG neuronal activity (as evidenced by *Zif268* and *c-Fos* immunohistochemistry) is silenced during HIWM training [28] and that ablation of adult neurogenesis (naturally occurring in this hippocampal sub-structure) is sufficient to boost performance in a similar task [46], we previously suggested that DG-dependent pattern separation function could lead to the generation of distinct memories, and thus PI, during HIWM training [28]. In order to test the hypothesis that pattern separation could induce the accumulation of PI in WM, we designed variants of our two behavioral tasks in which the need for pattern separation was lowered. In these new tasks, the arms of each pair were no longer adjacent (45° spaced) but formed a 90° angle, increasing the spatial difference between them. By using this paradigm making the discrimination of the arms easier for the rats, we expected an absence of performance drop as previously seen in HIWM training in experiment 1. We thus trained 26 rats in the LIWM90 or the HIWM90 tasks using a spaced training identical to the one used in experiment 1. The evolution of their performance is displayed in Fig 4. No significant difference between the performance of the two groups (no significant Group effect [F (1, 24) = 0.06340; p = 0.8033]) was observed in this condition. This result thus seems to confirm that the accumulation of PI is caused by the necessity to perform pattern separation in order to discriminate the arms of the maze. However, it can also be noted that rats trained in both the LIWM90 and HIWM90 tasks started on block 1 with a lower score (70%) than rats trained in the same tasks but with a 45° angle between choice arms (experiment 1, 80%). This may be due to the fact that in the 90° angle tasks rats take more time to associate extra-maze visual cues to each arm given that the spatial environment is larger (see Fig 1D). However, when the encoding of the distal visual cues is achieved after a few trials, the arms are more spatially discriminable with this 90° angle, explaining the absence of a PI effect at the end of training in the HIWM90 task.

## Discussion

Every day, every one of us is exposed to an important number of new information (i.e. concerning the daily weather, the meal we ate for lunch, a discussion with a colleague), but only a small part of this huge amount of information is pertinent enough to be consolidated into long-term memory. Many details are thus only temporarily useful (for instance, where we parked our car this morning) and need to be stored for a short-term period before being forgotten (yesterday’s parking spot being irrelevant and potentially interfering with the recall of our car’s location today). With this work, we precisely questioned the "short term period" of retention of information into WM: can WM content outlast its purpose by being stored more permanently?

We trained rats in classical DNMTP tasks in a radial maze. DNMTP and delayed-non-match-to-sample tasks are the most commonly used tasks to test WM in rodents but also in numerous species including humans [17,27,30]. We used a short delay of 15 seconds between the sample and the choice phase of each trial that corresponds to delays usually employed in such WM tasks [15]. The term “short-term memory” generally refers to the simple temporary storage of information, in contrast to WM, which implies a combination of storage and manipulation of this information [11,47]. Neuroimaging studies seem to show that perception, short-term and long-term memory are all performed in the same anatomical locations [48], and consequently WM has been defined as a cognitive process allowing a trace reactivated from long-term memory to be maintained in an unusually accessible state thanks to a limited-capacity attentional focus [49]. Of course, this retention and manipulation of information in the focus of attention cannot go unchallenged for a long period of time. Consequently, the retention of information into WM is not supposed to extend few seconds [50,51], but in that case, why not use the term “*working attention*” [52] to describe such brief retention of information? However, no consensus exists between researchers on a specific duration that qualifies as WM, beyond which other forms of memory must necessarily be inferred [17,18]. Moreover, if we go back to the above example of the parking spot, we face a conundrum. Frequently, the time for us to retrieve our car largely extends several seconds. In fact, it can expand to several hours, and in that case information related to this parking location could be thought of as being stored in long-term memory rather than WM. However, it seems odd to think that this kind of momentarily useful information can be considered stored in long-term memory. Obviously, such trivial information is not equivalent to the information pertaining to the last conference you attended or the name of your new colleague—information that would be preferable to keep as long as possible into long-term memory. Moreover, the information about the location of our car changes every time we use it and has no purpose to be permanently stored in memory (and therefore has to be stored temporarily). This is why some have suggested that WM is more a form of forgetting than a form of memory [22]. It is precisely to tackle this interesting link between WM and forgetting that we designed our two WM tasks, and we previously showed that unlike LIWM training, HIWM training would require forgetting of past trials [29].

In the present study, we showed that information supposedly forgotten after each trial during HIWM training were stored from one trial to another (during a delay longer than 15 seconds) and even from one day to the next, building PI and impairing rats' performance as we have previously shown [28,35,36]. In HIWM training, the use of cognitive resources in WM is thus no longer optimal because adaptive forgetting of information may not be sufficient to erase PI after a trial in this very repetitive task. During the first 5 days of training, the drop of performance previously observed by Roberts and Dale across trials [34] was present, even if it appeared only on the fourth and last daily trial in our HIWM group while Roberts and Dale showed this drop sooner (on the second trial) in their experiments. This difference may be explained by the differences existing between the protocols used in the two studies. During the sample phase, Roberts and Dale’s rats visited four arms in contrast to ours visiting only one arm. Therefore, Roberts and Dale’s procedure considerably increased not only the memory load (number of items to store during the within-trial delay) of the task, but also the delay itself between the presentation of the first of the four arms visited and its subsequent re-exposition during the choice phase. The differences between Robert and Dale’s tasks and ours could explain why the effect of PI is visible as soon as the second trial in one case, and later on (fourth trial) in the other. Nevertheless, no such drop was observed during LIWM training, suggesting that the level of PI is lower in this task than in the one used by Roberts and Dale. Moreover and interestingly, we showed that performance of HIWM rats decreased after 5 days of training, a result contrasting with the improvement of performance seen by Roberts and Dale after few days of training. We interpret this decrease in performance after five days of HIWM training as the consequence of an absence of memory reset [26] that would normally empty the content of WM between two days of training. Therefore, by making the task more repetitive than the one used by Roberts and Dale, we have managed with HIWM training to consolidate the content of WM into a more durable form of memory, generating PI deleterious to the performance of rats tested in this condition. However we do not know if the WM content more durably stored was still stored in WM (and in this case WM content lifespan would not be brief), or in an intermediary form of memory between WM and long-term memory. Nevertheless it would be surprising that this WM content is stored into long-term memory because information concerning passed trial are no longer useful, as it is the case with previous parking spots in our above example.

In their article, Roberts and Dale tried to dissect the PI effect by attempting to suppress it. Based on human studies [53,54], they assumed that PI disappears when trials are more temporally distinct. For them, PI arises from the difficulty for the rat to attribute "*temporal markers*" to the successive runs in the maze. In order to make trials more distinct in time, they increased the between-trial interval from 60 to 240 seconds, and in a second experiment they even removed the rat from the central platform of the maze during this interval. These two attempts failed, and PI still decreased the rats’ performance on the second trial of each session. In the present study, we made two different hypotheses about the building of PI that we believe are validated by our results. First, in experiment 2, we anticipated that spaced training could contribute to the consolidation of the content of WM (and thus in generating PI) from one day to the next. This assumption was confirmed as massed training concentrated in one single day did not alter performance of HIWM trained rats as compared to LIWM trained animals, a result suggesting that massed HIWM training does not produce PI detrimental to performance (as it is the case in spaced HIWM training—experiment 1). These results are in agreement with several learning theories attempting to account for the numerous evidences about the superiority of spaced learning over massed training [55–59]. According to the study-phase retrieval theory [44,60], while spaced training fosters reactivation of a memory trace formed by previous trials, such a memory trace is still active during massed training and is thus not reactivated and further reinforced. Paradoxically, the inefficacy of massed training for consolidation is beneficial when performing WM tasks during which a high level of PI is presented. In such tasks, one may believe that massed training prevents the consolidation of past trials, and thus the negative action of PI on an ongoing new trial. However, interestingly, a strong PI effect was visible at the beginning of the second day of massed training. This suggests that PI must have been stored and “accumulated” on day 1 of HIWM training in a "non active" form (inside WM or in an intermediary form of memory), even if this PI did not alter rats’ performance on this very first day of the experiment. The 24h interval between day 1 and day 2 was sufficient to consolidate this PI, producing a strong impairment on day 2 performance. The role of sleep occurring during this 24h delay may be of fundamental relevance in the consolidation of PI given that much evidence points towards a role of sleep, and in particular REM sleep, in memory consolidation [61–63]. Recently however, we have shown that performance of rats trained in a spaced HIWM task was positively correlated to an increase in slow wave sleep amount and slow wave activity [35], a result in agreement with theories attributing to these sleep stage and oscillations a role in the weakening of synaptic transmission [64]. While memory is supposed to depend on the strengthening of synapses involved in learning processes [65–67], spatial forms of forgetting have been shown to rely on the weakening (downscaling or depression) of hippocampal synaptic transmission [68]. As HIWM training is strongly dependent on such synaptic depression and on the forgetting of previously stored information [28,29], it is not surprising that such training is also linked to higher quantities of slow wave sleep and activity [35]. These recent results concerning the dual role of sleep in memory consolidation (presumably during REM sleep) and PI erasing (during slow-wave sleep) suggest that these two sleep-dependent processes could act in a competitive or antagonistic manner on PI processing. In experiment 1, with spaced training (sleep periods separating two training sessions), sleep could decrease PI at the beginning of training (leaving performance unaltered) by a slow-wave sleep-dependent process. After several days of training however, a REM sleep-dependent process could counteract such process, and consolidate PI (impairing performance). However, with massed training (experiment 2), slow-wave sleep-dependent decrease of PI may not be strong enough to prevent PI consolidation by a REM sleep-dependent process, leaving performance impaired at the beginning of day 2. Future work is required to confirm such hypothesis concerning the role of sleep in PI processing.

Several studies have shown that PI is linked to sample/choice perceptual discriminability [17,24,32,69]. A second question thus raised by our study was to ask if decreasing the need for pattern separation suppresses PI. Indeed, we showed that increasing spatial differences between arms of the pair tested in HIWM training (by increasing the angle formed by the pair of arms) was sufficient to alleviate the strong influence of PI on WM. Pattern separation is one essential feature of the DG [45], and we recently showed that HIWM training requires a very specific inactivation of this structure [28]. Conversely, we showed that a chronic treatment (with estradiol) which augments DG (c-Fos) activity maximizes PI effect in mice submitted to repeated training in a radial maze task of short-term memory relatively similar to the one used in the present study [70]. Interestingly, the DG is also one of the three brain regions that undergo adult neurogenesis, with new neurons being continuously integrated in the hippocampal network [71]. Various studies have demonstrated a link between DG neurogenesis and pattern separation. Clelland et al. have thus shown that ablation of DG neurogenesis impairs performance of mice in a radial maze DNMTP task when the need for pattern separation is high [43], while Sahay et al. increased the survival of adult-born neurons in the DG and subsequently observed an improvement of pattern separation to discriminate similar contexts after fear-conditioning [72]. These studies suggest that DG neurogenesis is necessary for pattern separation. In parallel, our team showed that ablation of neurogenesis in the hippocampus of adult mice improved their performance in a HIWM task very similar to the one used in the present study [46]. In consequence, our results link these studies by establishing a causal relationship between pattern separation and PI accumulation in the HIWM task. In the traditional HIWM task, and contrary to its variant HIWM90, the overlap between extra-maze visual cues associated to the two arms is extremely high (for all choice phases). Therefore, rats need to perform pattern separation at each choice phase in order to discriminate these arms. Performing pattern separation implies a storage mechanism generating neuronal traces with a lower overlap than the one present between the spatial localization of the arms in reality [73]. These neuronal representations would be consolidated during the interval between two days of training, and this consolidation would be enhanced by spaced training [44]. We can thus hypothesize that each time the rat needs to make a choice, these distinct neuronal representations might be involuntarily reactivated to discriminate the arms. However, it is well known that reactivation of a consolidated memory can make it labile again, and subject to a process called reconsolidation [74,75]. Therefore, we can imagine that when spatial memories for the two arms are reactivated on a new day of training, this creates new updates of these memories, possibly involving new neurons(in particular 3–5 week-old neurons that present a high degree of facilitation for long-term potentiation [76,77]). These multiple representations in the DG could create an adaptive advantage for the rat by generating multiple neuronal traces facilitating the recall *via* multiple paths [78]. This is certainly the case during LIWM training when neuronal traces are less reactivated as one given arm is only visited once a day. However, we can hypothesize that during HIWM training the constant use of the same pair of arms creates a massive accumulation of all these slightly different neuronal representations of the two arms, generating PI that impair performances. In such conditions, silencing neuronal population in the DG could be an adaptive response to suppress pattern separation preventing any further accumulation of PI, an hypothesis we have already tested and verified [28,46].

## Conclusions

With this study, we have successfully shown that the content of WM is not necessarily erased/forgotten after few seconds or even few minutes as previously hypothesized by the resetting theory [26]: either the content of WM is not as brief as previously suggested, or there may be another form of memory between WM and long-term memory where information from WM could be stored for several days. We thus show that information stored in WM can outlast their purpose by being consolidated for days, disrupting WM by creating PI; a fact one can easily verified in everyday life: who has not experienced such interfering recall of past information (such as a previously retained addition of numbers, or a past parking spot) when retrieval of newer information is required? Our results question the distinction between WM and long-term memory, all the more so as neuroimaging studies tend to show that these two processes are both executed in the same anatomical locations [48]. Moreover, just like it seems to be inseparable from long-term memory, WM may also be intertwined with sensory perceptions, given that sensory cortical areas are highly involved in WM [79]. Therefore the definition of WM as a single process with clear boundaries in terms of duration, items stored, or brain areas involved seems a bit outdated. In fact, in the literature, one can find several different examples of what is stored in WM [79–83], even if the general definition of WM as being a limited capacity system responsible for the transient holding and manipulation of information seems to be consensual [11]. The term WM was created at a time when scientists thought the mind as a complex computer [4]. We now know that this analogy is no longer relevant and nowadays, it is maybe more computer science that benefits from cognitive neuroscience by trying to recreate the mind in the machine. Although our data were collected in rodents, one can question the existence of WM in humans as well, and the distinction between what is memorized and what is forgotten is perhaps much more pertinent to the study of cognitive functions than the previously described division between long-term and WM. Alternatively, our study pertaining to spatial WM, one may hypothesize that our findings concerning the long-term duration of WM may not be generalized to all forms of WM and may be specific of spatial WM only. Future research will help clarifying this issue.

## Supporting information

S1 Table Recapitulative behavioral results. Results are expressed as a mean percentage of correct choices, SEM and number of animals for each training group (LIWM, HIWM, mLIWM, mHIWM, LIWM90 and HIWM90) and for each block of two sessions (or trial) in experiments 1, 2 and 3.
(PDF)
