# Supplementary material for: Long-term effects of interference on short-term memory performance in the rat
Source: PLoS One. 2017 Mar 13;12(3):e0173834. doi: 10.1371/journal.pone.0173834 (PMC5348021; doi:10.1371/journal.pone.0173834)
Supplement: S1 Table — Results are expressed as a mean percentage of correct choices, SEM and number of animals for each training group (LIWM, HIWM, mLIWM, mHIWM, LIWM90 and HIWM90) and for each block of two sessions (or trial) in experiments 1, 2 and 3. (PDF) [file pone.0173834.s001.pdf]

Experiment 1

Data from Fig 2A

Blocks of 2 sessions	*LIWM* Mean	SEM	n	*HIWM* Mean	SEM	n
B1	81,818	2,984	33	79,688	2,479	40
B2	85,227	2,460	33	80,625	2,236	40
B3	85,606	2,986	33	78,750	3,042	40
B4	87,121	2,903	33	70,000	3,221	40
B5	89,394	2,672	33	72,500	2,802	40

Data from Fig 2B

	Trials	*LIWM* Mean	SEM	n	*HIWM* Mean	SEM	n
Sessions 1 to 5	T1	78,788	3,250	33	78,500	3,153	40
	T2	84,242	3,109	33	83,500	2,854	40
	T3	89,091	2,476	33	85,000	2,931	40
	T4	86,667	2,562	33	73,500	3,679	40
Sessions 6 to 10	T1	88,485	3,142	33	70,500	3,922	40
	T2	84,242	3,109	33	70,000	3,721	40
	T3	88,485	3,374	33	79,000	2,860	40
	T4	86,667	3,553	33	70,500	3,358	40

Experiment 2

Blocks of 2 sessions	*mLIWM* Mean	SEM	n	*mHIWM* Mean	SEM	n
B1	72,321	6,030	14	76,786	5,051	14
B2	65,179	3,511	14	75,893	4,031	14
B3	74,107	6,481	14	68,750	5,521	14
B4	72,321	4,573	14	77,679	3,257	14
B5	81,250	3,876	14	83,036	4,062	14
B6	82,143	3,868	14	66,964	3,368	14
B7	86,607	3,812	14	66,071	6,206	14
B8	89,286	4,114	14	71,429	4,802	14
B9	94,643	2,159	14	79,464	3,614	14
B10	94,643	2,525	14	85,714	3,431	14

Experiment 3

Blocks of 2 sessions	*LIWM90* Mean	SEM	n	*HIWM90* Mean	SEM	n
B1	66,346	6,069	13	70,192	5,012	13
B2	68,269	5,769	13	74,038	5,382	13
B3	75,962	3,598	13	81,731	3,906	13
B4	74,038	4,578	13	74,038	4,578	13
B5	87,500	5,103	13	76,923	4,210	13
